# Resistin reinforces interferon λ-3 to eliminate hepatitis C virus with fine-tuning from RETN single-nucleotide polymorphisms

**DOI:** 10.1038/srep30799

**Published:** 2016-08-01

**Authors:** Ming-Ling Chang, Kung-Hao Liang, Cheng-Lung Ku, Chia-Chi Lo, Ya-Ting Cheng, Chen-Ming Hsu, Chau-Ting Yeh, Cheng-Tang Chiu

**Affiliations:** 1 Liver Research Center, Division of Hepatology, Department of Gastroenterology and Hepatology, Chang Gung Memorial Hospital, Taoyuan, Taiwan; 2 Department of Medicine, College of Medicine, Chang Gung University, Taoyuan, Taiwan; 3 Laboratory of Human Immunology and Infectious Diseases, Graduate Institute of Clinical Medical Sciences, Chang Gung University, Taoyuan, Taiwan

## Abstract

The effect of resistin (RETN) on the response to anti-HCV therapy remains unclear. A prospective cohort study was performed using 655 consecutive HCV patients, of whom 513 had completed a course of interferon-based therapy. Multivariate and GEE analyses revealed four RETN single-nucleotide polymorphisms (SNPs), rs34861192, rs3219175, rs3745367 and rs1423096, to be synergistically associated with resistin levels. After adjusting for co-factors such as interferon λ-3 (IFNL3)-rs12979860, the resistin level and the hyper-resistinemic genotype at the 4 RETN SNPs were positively and negatively associated with a sustained virological response (SVR), respectively. RETN-rs3745367 was in linkage disequilibrium with IFNL3-rs12979860. Compared to non-SVR patients, SVR patients had higher levels of pre-therapy resistin, primarily originating from intrahepatic lymphocytes, stellate cells, Kupffer cells, hepatic progenitor cells and hepatocytes. This difference diminished over the course of therapy, as only SVR patients exhibited a 24-week post-therapy decrease in resistin. Both resistin and IFNL3 mRNAs were upregulated, but only resistin mRNA was upregulated by recombinant resistin in peripheral blood mononuclear cells with and without hyper-resistinemic genotypes of the 4 RETN SNPs, respectively. Fine-tuned by RETN SNPs, intrahepatic, multi-cellular resistin reinforced IFNL3 in eliminating HCV via immunomodulation to counteract pro-inflammation. These results encourage the development of novel resistin-targeted anti-viral agents.

Hepatitis C virus (HCV), a human pathogen with variants classified into 7 major genotypes, is responsible for acute and chronic liver disease and infects an estimated 130–170 million individuals worldwide^1^. In addition to cirrhosis and hepatocellular carcinoma, HCV causes metabolic alterations, including hepatic steatosis, hypolipidemia, insulin resistance (IR), diabetes and obesity^2^,^3^,^4^. Specifically, HCV affects insulin signaling, and its life cycle is closely associated with host lipid metabolism^2^,^4^. Adipose tissue has emerged as an important endocrine organ due to its release of adipokines, which regulate lipid and glucose metabolism via the adipoinsular axis^5^,^6^. Although both HCV infection and adipokines are crucially involved in metabolism, no definitive causal relationship between HCV infection and adipokine alterations has been established to date^4^.

As a 12.5-kDa cysteine-rich adipokine, resistin (RETN) was discovered in a screen for adipocyte gene products downregulated by antidiabetic drugs^7^. Rodent resistin is primarily produced in adipocytes and reduces glucose uptake by tissues, thereby contributing to IR^8^. In contrast, human resistin is predominantly expressed by peripheral blood mononuclear cells (PBMCs), macrophages and bone marrow cells^9^. Additionally, the mouse and human resistins share only approximately 50% similarity at the genomic DNA, mRNA and amino acid levels^10^. Thus, the relevance of human resistin in IR has been challenged by the differences in origin and structure between the human and rodent resistins and by controversy in human epidemiological studies^11^. Instead, recent clinical data suggest that human resistin is pro-inflammatory and can serve as a biomarker of cardiovascular disease^12^. Based on the emerging interrelationship between metabolic and inflammatory diseases^13^, metabolism and inflammation may be linked by resistin activity, thereby affecting the disease process of hepatitis C. Approximately 70% of resistin expression is attributed to genetic effects, and notably, several single-nucleotide polymorphisms (SNPs) are highly correlated with resistin levels^14^,^15^. Therefore, a relationship between resistin alteration and HCV infection may not be revealed without adjusting for crucial genetic, metabolic, inflammatory and viral profiles.

Accordingly, we sought to elucidate cross-sectional and longitudinal relationships between HCV infection and resistin alteration after adjusting for crucial confounders in a prospective study of patients with chronic hepatitis C (CHC) before, during and after anti-HCV therapy. Immunological and genetic experiments were performed to assess the associated basis.

## Materials and Methods

### Patients

The study group consisted of subjects >18 yr with CHC, as defined by anti-HCV antibody (Ab) positivity and detectable HCV RNA for >24 weeks. We excluded subjects with human immunodeficiency virus, hepatitis B infection, hemochromatosis, coronary heart disease, malignancy or renal insufficiency and recipients of solid organ transplants. The control group consisted of subjects >18 yr who were selected based on the absence of major diseases, including CHC.

### Study design

As shown in Supplementary Figure 1, 840 consecutive patients positive for anti-HCV Ab (Abbott Laboratories, IL, USA) for more than 6 months were surveyed for participation in the current study at a tertiary referral center between July 2009 and June 2015. In total, 655 CHC patients were recruited, of whom 513 had completed a course of anti-HCV therapy with pegylated interferon α-2b (1.5 μg/kg/week) and ribavirin (800–1400 mg/day) for up to 24 or 48 weeks according to response-guided therapeutic criteria^3^. HCV RNA levels and genotypes were tested using COBAS Amplicor and InoLipa methods (Roche Diagnostics, Tokyo, Japan). SNPs of interferon λ-3 (IFNL3) or interleukin-28B-rs12979860 were assessed using genomic DNA, as described previously^3^. Preliminary screening of 10 RETN SNPs, including 4 in the promoter, 1 in the second intron, and 5 in the downstream region of RETN at 19p13.2 (rs1862513, rs10401670, rs34124816, rs3219175, rs3745367, rs3745369, rs1477341, rs4804765, rs1423096 and rs34861192)^14^,^15^, were assessed in 200 randomly selected patients of the 655 CHC patients using TaqMan SNP Genotyping Assays (Applied Biosystems, MA, USA) (Supplementary Table 1).

The following baseline factors were evaluated for all 655 patients: sex, age and body mass index (BMI); IFNL3-rs12979860 and RETN SNPs, including rs1423096, rs34861192, rs3745367 and rs3219175 genotypes; the HCV RNA level and HCV genotype; aspartate aminotransaminase (AST), alanine aminotransaminase (ALT), fasting glucose and insulin levels; homeostasis model assessment of insulin resistance (HOMA-IR); total cholesterol (TC), triglycerides (TGs), high-sensitivity C-reactive protein (hs-CRP), ferritin, thyroid-stimulating hormone (TSH), creatinine, complement component 3 (C3), and C4 levels; white blood cell (WBC) count and platelet count; the AST-to-platelet ratio index (APRI); the estimated glomerular filtration rate (GFR) calculated using the Cockcroft-Gault equation; and resistin levels (R&D Systems, MN, USA). For the 513 patients who had completed anti-HCV therapy, all the aforementioned factors were evaluated at 2 weeks before therapy, after 4, 12 and 24 weeks of therapy, at the end of therapy, and at 12 and 24 weeks after the end of therapy. IR was defined as a HOMA-IR score ≥2.5. SVR was defined as undetectable levels of HCV RNA at 24 weeks after therapy completion. All of the serum biochemistry and viral marker measurements were performed using routine automated techniques at the clinical pathology or liver research laboratories of the hospital. Twenty normal male controls were enrolled for PBMC stimulation testing and a fasting peripheral blood (PB) smear. Ten paraffinized normal liver samples were acquired from the tissue bank of the hospital to perform liver histology studies. For immunohistochemical (IHC) analyses, fasting PB smears and liver biopsy samples were acquired from 20 CHC patients with SVR and 20 sex- and age-matched CHC patients without SVR before anti-HCV therapy.

### IHC analyses of PB smears and liver samples

PB samples were directly smeared onto glass slides fixed in formaldehyde solution and stained using anti-resistin Ab (Santa Cruz Biotechnology, CA, USA). For liver samples, IHC analyses of resistin, cluster of differentiation 68 [CD68, marker of Kupffer cells (KCs), i.e., liver macrophages]^16^, CD43 (marker of lymphocytes)^17^, α-smooth muscle actin [marker of α-SMA-positive myofibroblasts, i.e., hepatic stellate cells (HSCs)]^16^ and cytokeratin-7 [CK-7, marker of hepatic progenitor cells (HPCs)]^18^ were performed using anti-resistin (Santa Cruz Biotechnology), anti-CD68, anti-CD43 (Dako, Glostrup, Denmark), anti-α-SMA (Sigma, Gillingham, Dorset) and anti-CK-7 (Proteintech Group, Inc., IL, USA) Abs, respectively. Serial sections of paraffinized liver samples were used according to the manufacturers’ protocols, as the nonparenchymal cell cytoplasm is insufficient for co-staining of resistin and cell markers in the same sections. Correlations for these proteins were assessed in parallel sections. The intensity of resistin expression by IHC was measured using ImageJ software (http://imagej.nih.gov/ij/, National Institutes of Health, USA).

### Functional assays between IFNL3 and resistin

PBMCs were isolated from 1 ml of whole blood from 20 normal subjects in triplicate using density gradient centrifugation with BD Vacutainer CPT heparin tubes. One day before the experiment, the PBMCs were plated at a density of 1 × 10^6^ cells/well in 24-well plates. After replacing the medium, recombinant resistin, IFNL3 proteins, and lipopolysaccharide (LPS) (R&D Systems, Minneapolis, MN) were added at a concentration of 100 ng/ml. As LPS induces resistin gene expression in macrophages^19^, it was used as a positive control. PBMCs from the same individuals not exposed to recombinant proteins served as the controls. The media were harvested in triplicate at 1, 2 and 4 hr after the recombinant proteins were added. The RNA levels of resistin and IFNL3 were assessed using quantitative reverse transcription real-time polymerase chain reaction (qRT-PCR). The primer sequences used for the qRT-PCR analysis of resistin and IL28B are listed in Supplementary Table 2.

### Statistics

All statistical analyses were performed using Statistical Product and Service Solutions software (SPSS package version 21, SPSS Inc., Chicago, USA). To compare different variables in two different groups, continuous variables were analyzed using Student’s *t*-tests; categorical variables were analyzed using chi-squared tests or Fisher’s exact tests, as appropriate. Groups of more than 2 were compared by one-way analysis of variance (ANOVA) for different variables, followed by post hoc tests. Univariate and multivariate analyses were used to determine relationships between the dependent and independent variables cross-sectionally, and generalized estimating equation (GEE) repeated measures tests were applied to determine relationships longitudinally. Paired *t*-tests were used to compare variables within individuals before and at 24 weeks after anti-HCV therapy. In addition to dominance-recessive mode analysis, for all SNPs evaluated, we consistently encoded the major homozygous genotype as 0, the heterozygous genotype as 1, and the minor homozygous genotype as 2^20^. Nonparametric statistics were applied as indicated. Hardy-Weinberg equilibrium and minor allele frequencies of the genotypes were determined to narrow down the 10 RETN SNPs. The statistical significance of the genotype correlations was evaluated using linear-by-linear association. Visualization of the correlation was achieved using generalized association plots^21^. The diagnostic accuracy of using pre-therapy resistin to predict therapeutic response was assessed in terms of true positive (sensitivity) versus true negative (1-specificity) responses using receiver operating characteristic (ROC) analyses. Statistical significance was defined at the 5% level based on two-tailed tests of the null hypothesis.

The study protocol conformed to the ethical guidelines of the 1975 Declaration of Helsinki and was approved by the institutional review board of the Chang Gung Memorial hospital. All subjects provided written informed consent to participate in this study.

## Results

### Four RETN SNPs, including rs34861192, rs3219175, rs3745367 and rs1423096, were synergistically associated with resistin levels

The baseline characteristics of the 655 CHC patients and comparisons between those with and without SVR are listed in Table 1. Among the 513 CHC patients who finished anti-HCV therapy, higher levels of pre-therapy resistin and platelet count, higher percentages of the IFNL3-rs12979860 CC genotype and HCV genotype 2, lower levels of HCV RNA, HOMA-IR and triglycerides, and lower percentages of HCV genotype 1 were found in those with SVR (n = 428) compared with those without SVR (n = 85) (Table 1).

**Figure 1:**
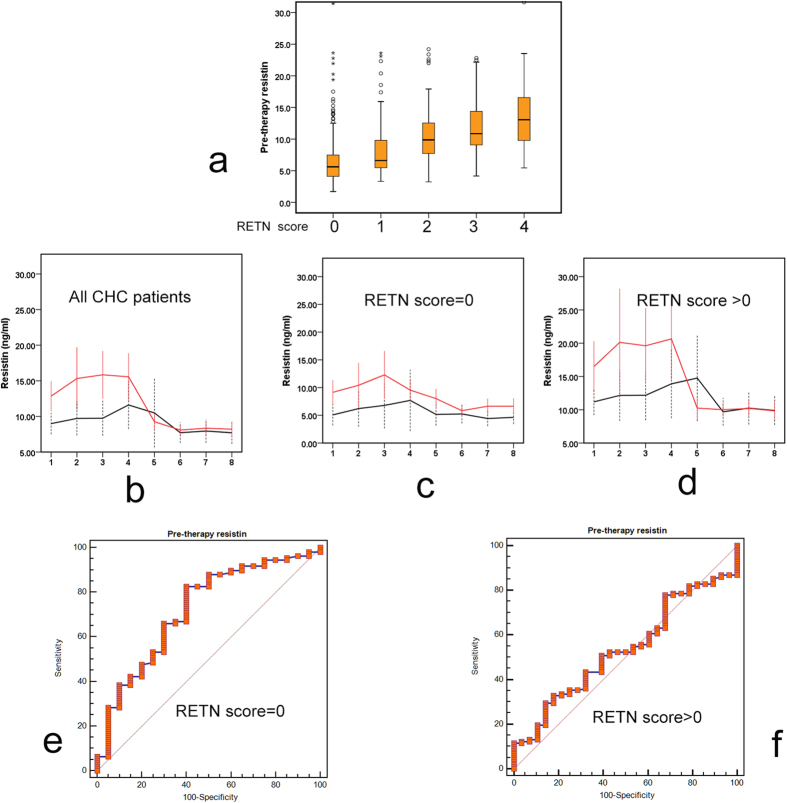
Resistin levels and receiver operating characteristic (ROC) analyses. (**a**) Box-and-whisker plots of pre-therapy resistin levels in chronic hepatitis C (CHC) patients according to resistin (RETN) scores. The outliers are shown as circles or stars. (**b**–**d**) Longitudinal trend of mean resistin levels (+/−95% confidence interval) in CHC patients according to RETN scores. Red lines: resistin levels of CHC patients with a sustained virological response (SVR); black lines: resistin levels of CHC patients without SVR. Blood draw time points: 1, 2 weeks before therapy; 2, after 4 weeks of therapy; 3, after 12 weeks of therapy; 4, after 24 weeks of therapy; 5, after 36 weeks of therapy; 6, after 48 weeks of therapy; 7, after 60 weeks of therapy; and 8, after 72 weeks of therapy. (**e**,**f**) ROC analyses of pre-therapy resistin levels in predicting SVR of CHC patients with a RETN score = 0 (e) or RETN score >0 (f).

Among the 10 RETN SNPs (Supplementary Table 1) investigated, the genotype distributions of one SNP (rs1862513) significantly deviated from Hardy-Weinberg equilibrium (*p* < 0.05) and were not further analyzed. Two SNPs (rs34124816 and rs4804765) have low minor allele frequencies in the Taiwanese population (<15%) and were de-prioritized for further analysis. Of the remaining 4 downstream SNPs, the genotypes correlated with each other (Pearson’s correlation > 0.3, *p* < 0.001). Thus, SNP rs1423096, which is the farthest SNP downstream and which did not overlap with the next gene (MCEMP1), was selected as a representative SNP. Consistently, the genotypes of the excluded 6 RETN SNPs did not correlate with pre-therapy resistin levels. In contrast, the genotypes of the 4 selected SNPs, rs34861192 and rs3219175 in the promoter, rs3745367 in the second intron, and rs1423096 (all *p* < 0.001) in the downstream region of RETN, correlated highly with pre-therapy resistin levels. As determined by post hoc tests, the “TT” and “TC” variants of rs1423096 (*p* < 0.001), “AA and GA” variants of rs34861192 (*p* < 0.001), “AA” variant of rs3745367 (*p* = 0.01), and “AA” and “GA” variants of rs3219175 (*p* < 0.001) showed higher pre-therapy resistin levels than the other variants of the same alleles. We stratified the patients into 5 groups according to RETN scores, which ranged from 0 to 4 and were defined by the number of the hyper-resistinemic genotypes among the 4 RETN SNPs (e.g., for rs1423096, hyper-resistinemic genotypes “TT” and “TC” were coded as “1”, and the normal resistinemic genotype “CC” was coded as “0”) (Supplementary Table 3). The 4 RETN SNPs had synergistic effects on the pre-therapy resistin levels, and RETN scores positively correlated with pre-therapy resistin levels (*p < *0.001) (Fig. 1a). Univariate and multivariate analyses showed that the RETN score, pre-therapy ferritin level, WBC count and GFR were independent factors of pre-therapy resistin levels (Table 2).

### The 4 RETN SNPs and SVR affected the longitudinal trend of the resistin level

For the 513 CHC patients who completed anti-HCV therapy, GEE analysis revealed that the RETN score, SVR, therapeutic intervention, ALT and APRI levels, and WBC count determined the longitudinal trend of the resistin level (Table 3).

### The 4 RETN SNPs and resistin were associated with SVR after adjustment for confounding factors, including the IFNL3 SNP genotype

As shown in Table 4, the IFNL3 SNP, HCV genotype, HCV RNA, pre-therapy TG and platelet levels were factors for SVR in our univariate analysis. BMI, the IFNL3 SNP, HCV genotype, RETN score, and pre-therapy resistin and ferritin levels were independently associated with SVR in multivariate analysis.

### Higher pre-therapy resistin levels were observed in CHC patients with SVR compared to those without, but the difference diminished over the course of therapy

Before anti-HCV therapy, patients with SVR had higher resistin levels than those without SVR (Table 1), a difference that gradually diminished: the resistin levels of those with SVR decreased over the course of treatment. In contrast, the resistin levels in those without SVR fluctuated only slightly during the course of treatment, regardless of RETN score stratification (Fig. 1b–d).

Paired *t*-test comparisons between pre- and post-therapy variants in the CHC patients who completed anti-HCV therapy showed that at 24-weeks post-therapy, the levels of ALT (99.0 + /−90.5 vs. 21.0 + /−14.6 U/L, *p* < 0.001), APRI (1.42 + /−1.60 vs. 0.48 + /−0.39, *p* < 0.001) and resistin (13.24 + /−17.18 vs. 8.19 + /−4.96 ng/ml, *p* < 0.001) decreased but that of TC (170.7 + /−31.9 vs.186.0 + /−35.7 mg/dL, *p* < 0.001) and TG (98.3 + /−43.2 vs. 115.9 + /−65.9 mg/dL, *p* < 0.001) levels increased in patients with SVR. In contrast, all of the aforementioned factors remained indifferent in patients without SVR after therapy.

Additionally, no differences in resistin level were noted between patients with or without IR, regardless of anti-HCV therapy (before anti-HCV therapy, *p* = 0.207; after anti-HCV therapy, *p* = 0.214).

### A pre-therapy resistin level > 3.956 ng/ml predicted SVR among patients with a RETN score of 0

To predict SVR, the RETN score was used to stratify a ROC analysis of pre-therapy resistin levels because it was associated positively with the resistin level (Tables 2 and 3) but negatively with the SVR rate in the multivariate analysis (Table 4). The area under the ROC curve (AUC) for patients with a RETN score of 0 was 0.745 (*p* = 0.001, sensitivity = 82.57%, specificity = 60.00%), with a cut-off level of 3.956 ng/ml for pre-therapy resistin in determining SVR (Fig. 1e). In contrast, for those patients with a RETN score >0, ROC analysis failed to provide a cut-off level of pre-therapy resistin in predicting SVR (RETN score >0, i.e., score 1–4, AUC: 0.534, *p* = 0.5404) (Fig. 1f).

### Higher rates of intrahepatic resistin-positive cells, including lymphocytes, HSCs, KCs, HPCs and hepatocytes, were observed in CHC patients with SVR than in those without SVR

**Figure 2:**
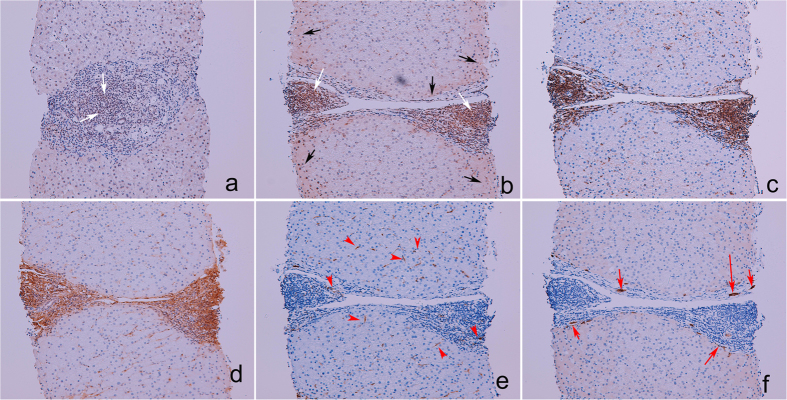
Immunohistochemical analyses of resistin and other proteins in the livers of chronic hepatitis C (CHC) patients before anti-hepatitis C viral therapy. (**a**) Resistin labeling in the liver from a CHC patient without sustained virological response (SVR). (**b**–**f**) Serial-section staining for resistin and other proteins in the liver from a CHC patient with SVR. (**a**,**b**) resistin staining. Clumps of resistin-positive nonparenchymal cells are labeled with white arrows; resistin-positive hepatocytes are labeled with black arrows. (**c**) Cluster of differentiation for 43(CD43) staining. (**d**) α-Smooth muscle actin staining. (**e**) CD68 staining. CD-68-positive cells (Kupffer cells) are labeled with red arrowheads. (**f**) Cytokeratin-7 staining. Cytokeratin-7-positive cells (hepatic progenitor cells) are labeled with red arrows.

Compared to normal controls, before anti-HCV therapy, the CHC patients had significantly higher PB (0.20 + /−0.10 vs. 48.2 + /−29.9%, *p* = 0.039) and hepatic (0.03 + /−0.01 vs. 25.88 + /−13.68%, *p* < 0.001) expression of resistin. Negligible resistin expression was observed in the livers of normal controls. Among the CHC patients, no correlation was observed between PB and intrahepatic resistin expression (*p* = 0.535) or between PB resistin expression and serum resistin levels (*p* = 0.654). However, a correlation was observed between intrahepatic resistin expression and serum resistin levels (*p* = 0.016); the patients with SVR had higher intrahepatic expression (42.8+/−26.9 vs. s6.7+/−2.3%, *p* = 0.012) but indifferent PB resistin expression (46.6+/−21.8 vs. 49.9+/−32.5%, *p* = 0.343) compared to those without SVR. As illustrated by serial sections from a representative case of a CHC patient with SVR, most intrahepatic resistin-positive cells were non-parenchymal cells (Fig. 2a,b, white arrows), including lymphocytes (Fig. 2c), HSCs (Fig. 2d), KCs (Fig. 2e) and HPCs (Fig. 2f). Some hepatocytes also expressed resistin (Fig. 2b, black arrows).

### All 4 analyzed RETN SNPs were in linkage disequilibrium (LD) with each other; RETN-rs3745367 was in LD with IFNL3-rs12979860

**Figure 3:**
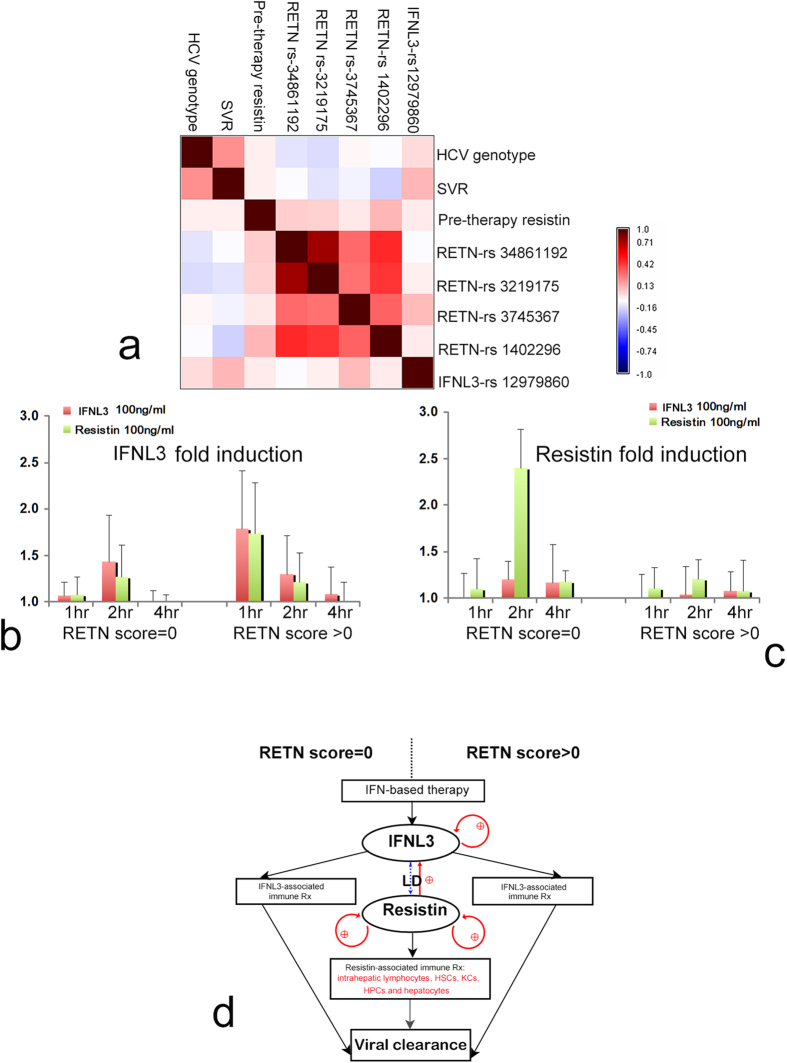
The association between resistin (RETN) and interferon λ-3 (IFNL3). (**a**) Pairwise correlations between genotypes and clinical parameters are shown in a color map. Spearman’s rank correlation coefficients are presented using a spectrum of colors ranging from dark brown to deep purple (the color bar is shown at the right). The HCV genotypes include genotype type 1 (coded as 1) and non-genotype type 1 (coded as 2). SVR: sustained viral response. SNPs are ordered by their locations along chromosome 19. The genotypes of 4 RETN SNPs are highly correlated with each other, forming a mosaic red-brown color block near the center of the color map. IFNL3 genotype “CC” is strongly correlated with RETN-rs3745367 genotype “AA” (Spearman correlation coefficient = 0.172, *p* = 0.013). (**b**,**c**) RNA fold changes for IFNL3 (**b**) and resistin (**c**) after stimulation with recombinant resistin (green bars) or IFNL3 (red bars) in peripheral blood mononuclear cells (PBMCs). (**d**) Proposed mechanism for the pathways associated with resistin and IFNL3 in interferon-based therapy stratified by RETN score. Panel to the left of the dashed line: pathways associated with RETN = 0; panel to the right of the dashed line: pathways associated with RETN >0. IFN: interferon; LD: linkage disequilibrium; Rx: reactions; HSCs: hepatic stellate cells; KCs: Kupffer cells; HPCs: hepatic progenitor cells; red arrows (straight or circle): upregulation. Please see the text for detailed descriptions.

Correlation tests showed the genotypes of the 4 aforementioned SNPs to be tightly correlated (all *p*-values for the correlation tests for any 2 of the 4 RETN SNP genotypes were <0.001). Furthermore, genotype “AA” of RETN-rs3745367 was associated with genotype “CC” of IFNL3-rs12979860 (linear-by-linear association *p* = 0.013, *D’* = 0.747) (Fig. 3a). These results indicate that all 4 RETN SNPs were in LD with each other and that RETN-rs3745367 was in LD with IFNL3-rs12979860.

### Resistin reinforced IFNL3 expression

The fold changes in IFNL3 (Fig. 3b) or resistin (Fig. 3c) mRNA expression in PBMCs stimulated with recombinant proteins compared to PBMCs from the same individuals without stimulation are shown with their associated *p*-values in Fig. 3 and Supplementary Table 4. For those with a RETN score > 0, both recombinant resistin (Supplementary Table 4, 1 hr, *p* < 0.001) and IFNL3 (Supplementary Table 4, 1 hr, *p* = 0.001; 2 hr, *p* = 0.002) significantly increased the expression of IFNL3 mRNA. Recombinant resistin also upregulated resistin mRNA expression (Fig. 3c and Supplementary Table 4, 2 hr, *p* = 0.002). For those with a RETN score of 0, recombinant resistin significantly upregulated resistin mRNA expression (Fig. 3c, Supplementary Table 4, 4 hr, *p* = 0.031).

## Discussion

To our knowledge, this prospective study is the first to demonstrate the favorable role and associated basis for resistin in the response to interferon-based anti-HCV therapy. The five most compelling results are described below. (1) Among the 10 RETN SNPs investigated, rs34861192, rs3219175, rs3745367, and rs1423096 were synergistically associated with resistin levels in CHC patients. (2) In addition to these 4 RETN SNPs, pre-therapy ferritin, WBC count and GFR level were associated with pre-therapy resistin levels, whereas SVR, therapeutic intervention, ALT and APRI levels, and WBC count were associated with the longitudinal trend in resistin levels. (3) After adjusting for other confounders, resistin levels were positively associated with SVR, whereas the number of hyper-resistinemic genotypes of the 4 RETN SNPs (ie. RETN score) was negatively associated. SVR patients had significantly higher pre-therapy resistin levels than non-SVR patients, and a pre-therapy resistin level >3.956 ng/ml predicted SVR in CHC patients with a RETN score of 0. (4) The differences in serum resistin level between SVR and non-SVR CHC patients mainly originated from intrahepatic lymphocytes, HSCs, KCs, HPCs, and hepatocytes. (5) Resistin may augment interferon-based anti-HCV therapy by reinforcing IFNL3. Genetically, RETN-rs3745367 was in LD with IFNL3-rs12979860. Functionally, both resistin and IFNL3 were upregulated, but only resistin was upregulated by recombinant resistin in PBMCs with RETN scores >0 and = 0, respectively.

HOMA-IR was not cross-sectionally or longitudinally associated with the resistin level in CHC patients, confirming that human resistin levels are not associated with glucose homeostasis but are associated with genetics (RETN SNPs)^14^,^15^, hepatic fibrosis/necroinflammation (ferritin)^22^, immunity (WBC counts)^23^, and renal function (GFR)^24^, even with HCV infection. Of note, only 4 of the 10 RETN SNPs documented in the literature^14^,^15^ were synergistically associated with resistin levels in CHC patients. Of great importance, hyper-resistinemia is favorable for SVR. Following anti-HCV therapy, only SVR patients exhibited post-therapy increases in TC and TG levels along with decreases in ALT, APRI, and resistin levels. These findings reflect an improvement in HCV-associated hypolipidemia^2^,^3^,^4^, hepatic necroinflammation and fibrosis subsequent to viral clearance. However, an improvement in hepatic necroinflammation cannot fully explain the decreased resistin levels, as the difference in resistin levels between the SVR and non-SVR patients vanished at 24 weeks post-therapy, a time when the non-SVR patients still suffered from virus-associated necroinflammation. These results suggest that resistin may not only be pro-inflammatory but also immunomodulatory, augmenting interferon-based therapy to clear HCV. The relatively low pre-therapy resistin levels in the non-SVR patients indicate the inadequate responsiveness of immunity during interferon-based therapy. For CHC patients without hyper-resistinemic genotypes for the 4 RETN SNPs, in addition to the IFNL3 “CC” genotype, pre-therapy resistin levels >3.956 ng/ml may serve as a feasible criterion for predicting favorable interferon-based therapeutic responses. Indeed, hyper-resistinemia in patients with CHC is consistently observed^25^,^26^, is reversed after viral clearance^27^, and causes moderate to-severe fibrosis^25^ but is thought not to be associated with therapeutic response^27^. The beneficial role of hyper-resistinemia in the response to interferon-based therapy could not be revealed without eliminating renal insufficiency, which profoundly increases resistin levels^24^, and without adjusting for RETN SNPs in over 500 CHC patients undergoing anti-HCV therapy.

The CHC patients exhibited higher expression of both PB and hepatic resistin than the controls. However, the difference in serum resistin levels between the SVR and non-SVR CHC patients primarily originated from the liver. This result highlights the importance of intrahepatic resistin in augmenting interferon-based anti-HCV efficacy and is consistent with the hepatotropic characteristics of HCV. All of the intrahepatic resistin-positive cells, including lymphocytes, HSCs, KCs, HPCs, and hepatocytes, are reported to express resistin^28^,^29^,^30^,^31^. Resistin may become available to resident hepatocytes through paracrine mechanisms via local production by lymphocytes, HSCs and KCs^30^,^32^, thereby accelerating hepatic necroinflammation and fibrosis^33^. Consistently, resistin levels correlate with inflammatory markers in chronic liver disease^34^ and unfavorable outcomes in non-septic critical patients^35^. In the current study, longitudinal resistin levels correlated with APRI, and both HSCs and HPCs expressed resistin. HPC activation occurs in response to hepatic oxidative stress and is associated with fibrosis^31^, as evidenced by HSC involvement. Altogether, the pro-inflammatory activities of resistin are harmful if left untreated, particularly with respect to accelerating hepatic fibrosis. In addition, viral clearance following interferon-based therapy is associated with the induction of a vigorous T-helper response^36^. KCs, HSC, and hepatocytes may serve as liver antigen-presenting cells (APCs) to induce primary T-cell activation^37^. Resistin is upregulated during monocyte–macrophage differentiation^7^ and induces NF-kB activity in mature T-helper cells, thereby enhancing cell chemotaxis^38^. Thus, the fact that KCs, lymphocytes, HSCs, and hepatocytes all expressed resistin suggests that resistin might initiate innate immunity with macrophage activation to invoke adaptive immunity, whereby a reinforcing T-helper response boosts the efficacy of interferon-based therapy. In addition, IFNL3 is expressed predominantly in APCs and is upregulated by interferons^39^, and IFNL3 SNP genotypes are highly associated with the efficacy of interferon-based therapy in CHC^40^. However, IFNL3 SNPs do not affect expression of the IFNL3 gene itself, and the link between an IFNL3 SNP-associated therapeutic response and IFNL3 expression remains elusive^41^. The well-established LD between RETN and IFNL3 SNPs in the present study indicates that the aforementioned elusive link may be related to complicated genetic accommodation^42^. Namely, two correlated genes, RETN and IFNL3, might together modulate host immunity against HCV. Moreover, functional connections between IFNL3 and resistin were identified. Both resistin and IFNL3 mRNAs were upregulated, but only resistin mRNA was upregulated by recombinant resistin in PBMCs with and without hyper-resistinemic genotypes of the 4 RETN SNPs, respectively. In effective IFNL3 reinforcement, more benefits appeared to be obtained by the CHC patients with RETN hyper-resistinemic genotypes than without. However, this benefit might be compromised or even reversed in viral clearance, as the presence of hyper-resistinemic genotypes suggest aggravated hepatic necroinflammation/fibrosis subsequent to high baseline resistin levels. The deteriorated milieu of hepatic necroinflammation, such as severe hepatic fibrosis, is actually detrimental for HCV clearance^43^. This likely accounts for why pre-therapy resistin levels failed to predict the response rate among those with RETN hyper-resistinemic genotypes, and RETN score was negatively associated with SVR. The proposed basis of the connection between IFNL3 and resistin and associated anti-viral pathways is shown in Fig. 3d.

In an era in which most HCV infections can be eradicated with potent, direct-acting anti-viral agents^44^, the significance of RETN SNPs and resistin is still important in low-resource settings, where interferon-based therapy remains the mainstay treatment for CHC patients. Moreover, such findings may be extended for evaluating or improving therapeutic efficacy for other viral infections such as hepatitis B virus, for which interferon-α and nucleoside/nucleotide analogs are currently available but unsatisfactory^45^. In particular, interferon-α is effective in only approximately 30% of chronic hepatitis B cases^46^. Regardless, any anti-viral strategy targeting resistin-associated pathways demands extreme caution due to the double-edged nature of resistin, namely, necroinflammation and immunomodulation.

Taken together, the 4 RETN SNPs, rs34861192, rs3219175, rs3745367 and rs1423096, were synergistically associated with resistin levels. Fine-tuned by the 4 RETN SNPs, pre-therapy resistin levels positively affected the response to interferon-based anti-HCV therapy. The liver is the main source of resistin in the multi-cellular orchestration of viral clearance. Reinforcement of IFNL3 by resistin was evident at both the genetic and functional levels. This discovery of the beneficial role of resistin in the interferon-based anti-HCV therapeutic response encourages the development of novel anti-viral agents targeting resistin-associated pathways.

## Additional Information

**How to cite this article**: Chang, M.-L. *et al*. Resistin reinforces interferon λ-3 to eliminate hepatitis C virus with fine-tuning from RETN single-nucleotide polymorphisms. *Sci. Rep.* **6**, 30799; doi: 10.1038/srep30799 (2016).

## Supplementary Material

Supplementary Information

## Figures and Tables

**Table 1:** Baseline characteristics of the 655 patients with chronic hepatitis C before anti-hepatitis C virus (HCV) therapy.

Variants	Total (n = 655)	CHC patients completed anti-HCV therapy (n = 513)	SVR (+) (*n* = 428)	SVR (−) (*n* = 85)	*P* values between SVR(+) and SVR (+)
Male, n (%)	407 (62.1)	287 (55.9)	244 (57)	43 (50.5)	0.400
Age (yr)	55.1+/−11.9	54.1+/−11.5	53.8+/−11.6	56.0+/−10.9	0.124
BMI	24.9+/−3.7	25.0+/−3.5	24.8+/−3.6	25.6+/−4.0	0.069
IFNL3 or IL28B rs12979860 CC genotype (1/0)	565 (86.3)	435 (84.7)	378 (88.3)	57 (67.1)	0.006*
HCV genotype, n (%)					
G1	352 (53.7)	269 (52.4)	203 (47.4)	66 (77.6)	<0.001*
G2	267 (40.7)	222 (43.3)	206 (48.1)	16 (18.8)	<0.001*
G3	13 (1.98)	7 (1.36)	7 (1.6)	0 (0)	
G6	9 (1.3)	7 (1.36)	4 (0.9)	3 (3.5)	
Mixed type: G1 + G2; G1 + G3	7 (1);1 (0.1)	5 (0.97)	5 (1.16)	0 (0)	
Unidentified:	6 (0.9)	3 (0.58)	3 (0.61)	0 (0)	
HCV RNA (log_10_IU/mL)	5.97+/−1.11	5.95+/−1.14	5.84+/−1.18	6.47/+/−0.74	0.001*
Hyper-resistinemic genotype of RETN SNPs, n (%)					
rs1423096 TT or TC	235 (35.9)	189 (36.8)	147 (34.3)	42 (49.4)	0.06
rs34861192 AA or GA	231(35.2)	176 (34.3)	139 (32.4)	37 (43.5)	0.146
rs3745367 AA	136 (20.8)	111 (21.6)	90 (21.0)	21 (24.7)	0.58
rs3219175 AA or GA	226 (34.5)	178 (34.7)	146 (34.1)	32 (37.6)	0.581
RETN score #	1.29+/−1.47	1.26+/−1.55	1.24+/−1.43	1.59+/−1.63	0.167
Resistin (ng/ml)	10.95+/−12.83	11.89+/−14.99	12.53+/−16.61	8.79+/−5.42	0.004*
HOMA-IR	3.20+/−4.60	3.14+/−5.04	2.88+/−4.81	4.54+/−5.97	0.045*
TC (mg/dL)	171.1+/−34.1	171.5+/−32.3	171.4+/−35.8	172.4+/−30.1	0.788
TGs (mg/dL)	102.4+/−51.3	103.0+/−49.2	100.3+/−11.4	117.4+/−67.4	0.045*
ALT (U/L)	95.3+/−91.8	95.5+/−86.1	98.3+/−89.4	81.1+/−65.1	0.141
hsCRP (mg/L )	1.72+/−3.57	1.59+/−2.64	1.59+/−2.76	1.62+/−2.01	0.942
APRI	1.66+/−1.94	1.53+/−1.68	1.48+/−1.64	1.79+/−1.87	0.164
Ferritin (ng/ml)	350.2+/−404.3	360.2+/−408.9	346.3+/−406.8	424.4+/−430.7	0.252
WBC count (10^3^/uL)	5.68+/−1.9	5.77+/−1.89	5.79+/−1.91	5.67+/−1.76	0.628
Platelet (10^3^/uL)	176.5+/−64.7	178.4+/−59.5	183.0+/−58.5	155.7+/−59.4	0.001*
C3 (mg/dL)	105.8+/−20.6	106.4+/−19.3	107.1+/−19.48	102.8+/−17.78	0.148
C4 (mg/dL)	20.1+/−7.8	20.1+/−7.4	20.32+/−7.36	18.86+/−7.36	0.186
GFR (ml /min/1.73 m^2^)	92.6+/−32.5	102.7+/−33.5	103.9+/−31.6	101.2+/−34.8	0.759
TSH (uU/mL)	1.98+/−2.74	1.87+/−1.26	1.84+/−1.29	1.98+/−1.1	0.436

SVR: sustained virological response; BMI: body mass index; IFNL3 or IL28B: interferon-λ3 or interleukin 28B; G: genotype; log: logarithmic; SNPs: single-nucleotide polymorphisms; HOMA-IR: homeostasis model assessment-estimated insulin resistance: TC: total cholesterol; TGs: triglycerides; ALT: alanine aminotransferase; hsCRP: high sensitivity C- reactive protein; APRI: aspartate aminotransferase to platelet ratio index. WBC: white blood cells; C3: complement component 3; C4: complement component 4; GFR: glomerular filtration rate; TSH: thyroid-stimulating hormone; #numbers of hyper-resistinemic genotypes of the 4 RETN SNPs including rs1423096, rs34861192, rs3745367 and rs3219175. *p < 0.05.

**Table 2:** Univariate and multivariate analyses of pre-therapy variants used to determine the pre-therapy resistin levels in 655 patients with chronic hepatitis C.

Variants	Univariate analysis: 95% CI of estimated beta	Univariate analysis: *p* values	Multivariate analysis: 95% CI of estimated beta	Multivariate analysis: *p* values
Gender (female)	−0.366 ~ 4.35	0.097	−3.215 ~ 1.548	0.548
Age (yr)	−0.128 ~ 0.109	0.84	−0.197 ~ 0.008	0.072
BMI	−0.358 ~ 0.348	0.978	−0.571 ~ 0.016	0.063
IFNL3 or IL28B rs12979860 CC genotype (1/0)	−2.133 ~ 0.685	0.302	−4.533 ~ 1.728	0.378
HCV genotype	−0.721 ~ 1.473	0.501	−1.042 ~ 1.513	0.715
HCV RNA (log_10_IU/mL)	−0.299 ~ 0.007	0.061	−0.123 ~ 0.155	0.819
RETN score #	4.392 ~ 10.777	<0.001*	1.355 ~ 2.842	<0.001*
HOMA-IR	−0.328 ~ 0.207	0.656	−0.575 ~ 0.533	0.941
TC (mg/dL)	−0.084 ~ −0.003	0.033*	−0.07 ~ 0.002	0.066
TGs (mg/dL)	−0.038 ~ 0.016	0.44	−0.027 ~ 0.031	0.888
ALT (U/L)	−0.01 ~ 0.02	0.495	−0.03 ~ 0.004	0.123
hsCRP (mg/L )	−0.429 ~ 0.638	0.7	−0.336 ~ 0.409	0.845
APRI	−0.089 ~ 1.363	0.086	−0.716 ~ 1.454	0.503
Ferritin (ng/ml)	0.003 ~ 0.009	<0.001*	0.002 ~ 0.009	0.001*
WBC count (10^3^/uL)	0.083 ~ 1.495	0.029*	0.335 ~ 1.595	0.003*
Platelet (10^3^/uL)	−0.02 ~ 0.018	0.882	−0.31 ~ 0.022	0.744
C3 (mg/dL)	−0.76 ~ 0.058	0.793	−0.084 ~ 0.006	0.728
C4 (mg/dL)	−0.186 ~ 0.185	0.996	−0.138 ~ 0.198	0.796
GFR (ml /min/1.73 m^2^)	−0.13 ~ −0.054	<0.001*	−0.062 ~ −0.002	0.037*
TSH (uU/mL)	−0.105 ~ 1.618	0.085	−0.654 ~ 1.363	0.489

BMI: body mass index; IFNL3 or IL28B: interferon-λ3 or interleukin 28B; log: logarithmic; RETN: resistin; HOMA-IR: homeostasis model assessment-estimated insulin resistance: TC: total cholesterol; TGs: triglycerides; ALT: alanine aminotransferase; hsCRP: high sensitivity C- reactive protein; APRI: aspartate aminotransferase to platelet ratio index; WBC: white blood cells; C3: complement component 3; C4: complement component 4; GFR: glomerular filtration rate; TSH: thyroid- stimulating hormone. #numbers of hyper-resistinemic genotypes of the 4 RETN SNPs including rs1423096, rs34861192, rs3745367 and rs3219175. *p < 0.05.

**Table 3:** Generalized estimating equation analysis results used to determine the longitudinal trends of the resistin levels of the 513 patients with chronic hepatitis C who had completed the anti-hepatitis C virus (HCV) therapy.

Variants	Estimated Exp (B)	95% CI of estimated Exp (B)	*P* values
Gender (female)	1.844	0.178 ~ 19.892	0.608
Age (yr)	1.001	0.912 ~ 1.098	0.989
BMI	0.874	0.606 ~ 1.26	0.47
IFNL3 or IL28B rs12979860 CC genotype (1/0)	0.971	0.293 ~ 3.217	0.961
HCV genotype	0.643	0.143 ~ 2.898	0.565
HCV RNA (log_10_IU/mL)	1.009	0.933 ~ 1.091	0.823
RETN score #	13.904	5.699 ~ 33.921	< 0.001*
Therapy intervention (without)	0.018	0.001 ~ 0.288	0.005*
Therapy duration	0.821	0.599 ~ 1.125	0.22
SVR (0)	0.029	0.003 ~ 0.294	0.003*
HOMA-IR	0.972	0.856 ~ 1.103	0.659
TC (mg/dL)	0.959	0.92 ~ 1.00	0.051
TGs (mg/dL)	1.008	0.995 ~ 1.02	0.235
ALT (U/L)	0.988	0.978 ~ 0.998	0.017*
hsCRP (mg/L )	1.068	0.883 ~ 1.291	0.501
APRI	1.000	1.000 ~ 1.001	0.03*
Ferritin (ng/ml)	1.001	1.000 ~ 1.003	0.076
WBC count (10^3^/uL)	2.263	1.075 ~ 4.765	0.032*
Platelet (10^3^/uL)	0.954	0.89 ~ 1.024	0.191
C3 (mg/dL)	1.033	0.977 ~ 1.091	0.257
C4 (mg/dL)	0.937	0.85 ~ 1.032	0.186
GFR (ml /min/1.73 m^2^)	0.984	0.942 ~ 1.027	0.459
TSH (uU/mL)	1.001	0.993 ~ 1.009	0.778

95% CI of estimated Exp(B). BMI: body mass index; IFNL3 or IL28B: interferon-λ3 or interleukin 28B; log: logarithmic; RETN: resistin; HOMA-IR: homeostasis model assessment-estimated insulin resistance: TC: total cholesterol; TGs: triglycerides; ALT: alanine aminotransferase; hsCRP: high sensitivity C- reactive protein; APRI: AST to platelet ratio index; WBC: white blood cells; C3: complement component 3; C4: complement component 4; GFR: glomerular filtration rate; TSH: thyroid- stimulating hormone. #numbers of hyper-resistinemic genotypes of the 4 RETN SNPs including rs1423096, rs34861192, rs3745367 and rs3219175. *p < 0.05.

**Table 4:** Univariate and multivariate logistic analyses of pre-therapy variants used to determine the presence of sustained virological response (SVR) in 513 patients with chronic hepatitis C who had completed the anti-hepatitis C virus (HCV) therapy.

Variants	Univariate analysis 95% CI of OR (OR)	*P* values of Univariate analysis	Multivariate analysis 95% CI of OR (OR)	*P* values of multivariate analysis
Gender (female)	0.489 ~ 1.329 (0.807)	0.399	0.614 ~ 7.415 (2.133)	0.233
Age (yr)	0.961 ~ 1.005 (0.983)	0.125	0.917 ~ 1.032 (0.973)	0.363
BMI	0.88 ~ 1.005 (0.941)	0.07	0.715 ~ 0.979 (0.837)	0.026*
IFNL3 or IL28B rs12979860 CC genotype (1/0)	1.728 ~ 7.66 (3.639)	0.001*	1.91 ~ 32.93 (7.914)	0.004*
HCV genotype	2.458 ~ 8.768 (4.643)	<0.001*	1.637 ~ 34.904 (7.559)	0.001*
HCV RNA (log_10_IU/mL)	0.389 ~ 0.719 (0.529)	<0.001*	0.428 ~ 1.468 (0.792)	0.46
RETN score #	0.699 ~ 1.048 (0.856)	0.132	0.237 ~ 0.786 (0.431)	0.006*
Resistin (ng/ml)	0.992 ~ 1.075 (1.033)	0.113	1.065 ~ 1.509 (1.268)	0.008*
HOMA-IR	0.909 ~ 1.044 (0.955)	0.074	0.614 ~ 1.005 (0.785)	0.054
TC (mg/dL)	0.991 ~ 1.007 (0.999)	0.799	0.985 ~ 1.023 (1.004)	0.707
TGs (mg/dL)	0.989 ~ 0.999 (0.994)	0.01*	0.985 ~ 1.014 (0.999)	0.94
ALT (U/L)	0.999 ~ 1.007 (1.003)	0.144	1.000 ~ 1.028 (1.014)	0.05
hsCRP (mg/L )	0.899 ~ 1.103 (0.996)	0.942	0.733 ~ 1.048 (0.876)	0.148
APRI	0.788 ~ 1.042 (0.906)	0.168	0.513 ~ 1.436 (0.858)	0.561
Ferritin (ng/ml)	0.999 ~ 1.000 (1.000)	0.259	0.996 ~ 1.00 (0.998)	0.014*
WBC count (10^3^/uL)	0.896 ~ 1.201 (1.037)	0.627	0.599 ~ 1.224 (0.856)	0.395
Platelet (10^3^/uL)	1.003 ~ 1.013 (1.008)	0.001*	0.994 ~ 1.026 (1.010)	0.242
C3 (mg/dL)	0.996 ~ 1.028 (1.012)	0.144	0.997 ~ 1.087 (1.041)	0.065
C4 (mg/dL)	0.987 ~ 0.172 (1.028)	0.186	0.978 ~ 1.014 (0.997)	0.147
GFR (ml /min/1.73 m^2^)	0.99 ~ 1.007 (0.999)	0.758	0.98 ~ 1.014 (0.997)	0.715
TSH (uU/mL)	0.752 ~ 1.131 (0.922)	0.436	0.66 ~ 1.84 (1.102)	0.711

OR: odds ratio; BMI: body mass index; IFNL3 or IL28B: interferon-λ3 or interleukin 28B; log: logarithmic; RETN: resistin; HOMA-IR: homeostasis model assessment-estimated insulin resistance: TC: total cholesterol; TGs: triglycerides; ALT: alanine aminotransferase; hsCRP: high sensitivity C- reactive protein; APRI: AST to platelet ratio index; WBC: white blood cells; C3: complement component 3; C4: complement component 4; GFR: glomerular filtration rate; TSH: thyroid- stimulating hormone. #numbers of hyper-resistinemic genotypes of the 4 RETN SNPs including rs1423096, rs34861192, rs3745367 and rs3219175. *p < 0.05.
