# Supplementary material for: Resistin reinforces interferon λ-3 to eliminate hepatitis C virus with fine-tuning from RETN single-nucleotide polymorphisms
Source: Sci Rep. 2016 Aug 1;6:30799. doi: 10.1038/srep30799 (PMC4967850; doi:10.1038/srep30799)
Supplement: Supplementary Information [file srep30799-s1.pdf]

Resistin reinforces interferon λ-3 to eliminate hepatitis C virus with fine-tuning from RETN single nucleotide polymorphisms

Ming-Ling Chang, Kung-Hao Liang, Cheng-Lung Ku, Chia-Chi Lo, Ya-Ting Cheng, Chen-Ming Hsu, Chau-Ting Yeh, Cheng-Tang Chiu

Supplementary Figure 1. Flow chart for the enrollment of patients with chronic hepatitis C. HCV: Hepatitis C virus.

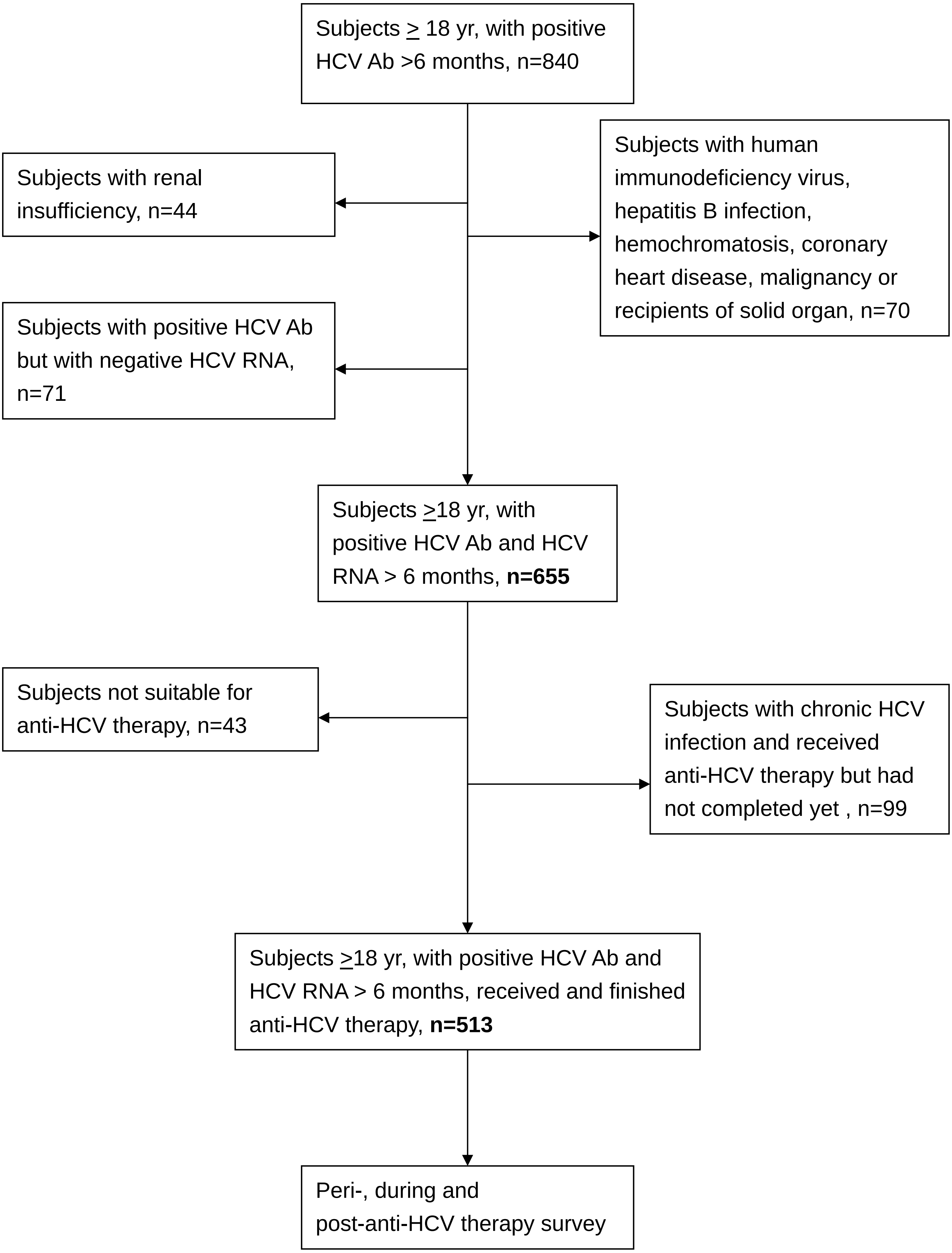

Supplementary Table 1. The 10 single-nucleotide polymorphisms (SNPs) evaluated in the pilot study. Among them, the 4 SNPs selected for the subsequent analysis were shown in bold face.

dbSNP ID	ID number of TaqMan assays	Chromosome	Location	MAF	HW#	Location in relation to RETN
**rs34861192**	AHBKFNB	19p13.2	7668688	0.21	0.75	promoter
rs34124816	C__64386062_10	19p13.2	7668789	0.12	0.43	promoter
rs1862513	C__1394112_10	19p13.2	7668906	0.35	0.03	promoter
**rs3219175**	C__27472440_10	19p13.2	7668968	0.21	0.68	promoter
**rs3745367**	C__1394113_10	19p13.2	7669624	0.39	0.96	intron
rs3745369	C__11510413_30	19p13.2	7670588	0.39	0.71	downstream
rs1477341	C__8351534_10	19p13.2	7671912	0.45	0.98	downstream
rs4804765	C__1394116_10	19p13.2	7672953	0.04	0.92	downstream
**rs1423096**	C__1394117_20	19p13.2	7674290	0.20	0.84	downstream
rs10401670	C__1394125_10	19p13.2	7677915	0.49	0.72	downstream, and overlap with the MCEMP1 gene

MAF: minor allele frequency

# P value of the Chi-square test on the Hardy-Weinberg equilibrium

Supplementary Table 2. Primer sequences used in the quantitative real time polymerase chain reactions of *resistin* (*RETN*), *interferon λ-3* (*IFNL3*), RNA 185S and beta-actin (ACTB). RNA 185S and ACTB served as internal controls.

**Genes**	**Primers**	**Sequences (5'->3')**
RETN	Hs-RETN_F	ACCACATGTCACTGCCAG
	Hs-RETN_R	AGGTTTATTTCCAGCTCCCC
RNA18S5	Hs-18s-rRNA_F	GAGACTCTGGCATGCTAACTAG
	Hs-18s-rRNA_R	GGACATCTAAGGGCATCACAG
IFNL3	hsa-IFNL3-F	AAGGACTGCAAGTGCCGCT
	hsa-IFNL3-R	GCTGGTCCAAGACATCCC
ACTB	Hs-ACTB_F	ACCTTCTACAATGAGCTGCG
	Hs-ACTB_R	CCTGGATAGCAACGTACATGG

Supplementary Table 3. The points for *RETN* score which employs the sum of the hyper-resistinemic genotypes of the 4 RETN SNPs.

RETN SNP ID	0 points	1 points
rs34861192	GG	AA or GA
rs3219175	GG	AA or GA
rs3745367	GG or GA	AA
rs1423096	CC	TT or TC

Supplementary Table 4. Fold changes and associated *p*-values of interferon λ-3 (IFNL3) or resistin mRNAs in the peripheral blood mononuclear cells (PBMCs) with exposure to recombinant proteins (resistin or IFNL3) compared to the IFNL3 or resistin mRNA of the PBMCs of the same individuals without exposure to recombinant proteins. *: $p<0.05$.

Recombinant proteins	mRNA checked/ time points	RETN=0, n=10		RETN>0, n=10	
		Fold changes of mRNAs in PBMCs with recombinant proteins to mRNAs in PBMCs without recombinant proteins	*p* values of fold changes of mRNAs between PBMCs with and without recombinant proteins	Fold changes of mRNAs in PBMCs with recombinant proteins to mRNAs in PBMCs without recombinant proteins	*p* values of fold changes of mRNA between PBMCs with and without recombinant proteins
IFNL3	IFNL3/1hr	1.14+/-0.42	0.234	1.78+/-0.89	0.01*
	IFNL3/2hr	2.11+/-2.52	0.137	1.29+/-0.55	0.02*
	IFNL3/4 hr	0.96+/-0.38	0.764	1.08+/-0.49	0.455
resistin	IFNL3/1hr	1.09+/-0.41	0.444	1.73+/-0.93	<0.01*
	IFNL3/2hr	2.39+/-4.08	0.243	1.21+/-0.53	0.083
	IFNL3/4 hr	0.797+/-0.35	0.062	0.91+/-0.49	0.413
IFNL3	Resistin/1hr	0.974+/-0.34	0.786	0.99+/-0.38	0.958
	Resistin/2hr	1.15+/-0.38	0.185	1.03+/-0.42	0.699
	Resistin/4 hr	1.12+/-0.42	0.304	1.07+/-0.31	0.31
resistin	Resistin/1hr	1.14+/-0.04	0.233	1.10+/-0.34	0.186
	Resistin/2hr	2.91+/-4.01	0.111	1.20+/-2.69	0.02*
	Resistin /4 hr	1.17+/-0.25	0.03*	1.07+/-0.41	0.414
